# Editorial: Viruses threatening stable production of cereal crops

**DOI:** 10.3389/fmicb.2015.00470

**Published:** 2015-05-19

**Authors:** Nobuhiro Suzuki, Takahide Sasaya, Il-Ryong Choi

**Affiliations:** ^1^Institute of Plant Science and Resources, Okayama UniversityKurashiki, Japan; ^2^Kyushu Okinawa Agricultural Research Center, National Agriculture and Food Research OrganizationKoshi, Japan; ^3^Plant Breeding, Genetics, and Biotechnology Division, International Rice Research InstituteLos Baños, Philippines

**Keywords:** cereal, rice, phytoreovirus, tenuivirus, luteovirus, polerovirus, insect vector

Between 2005 and 2008 the prices of rice, wheat and maize were more than doubled (von Braun, 2008). The sudden surge in cereal prices led or was led by major uncertainties to secure staple food supplies in many developing countries, especially in Asia. Several economic and social factors collectively appear to have triggered the unstable supplies of cereal crops during the recent food crisis (Headey and Fan, 2008). Besides, lower cereal crop productivities due to unfavorable environments and outbreaks of pests and diseases may have been associated, at least in part, with the unstable supplies of cereals during the period. In fact, massive outbreaks of brown planthoppers (BPH) transmitting rice viruses were reported in many areas of Indochina Peninsula during 2006 and 2008, which resulted in a significant decrease in international trade of rice (Ta et al., 2013). This research topic consists of four original research and seven review articles covering various aspects of cereal viruses and their vector insects that are considered as current or potential threats to stable production of cereal crops. We also hope that this e-book can provide the readership an update on the recent studies on a variety of cereal viruses.

The first two articles described two cereal viruses whose genome sequences were determined recently. Southern rice black-streaked dwarf virus (SRBSDV) is a new virus found in China in 2001. SRBSDV can infect maize, rice, and other monocotyledonous plants, and quickly spread to wide areas of China and Vietnam. SRBSDV is tentatively classified in the genus *Fijivirus* of the family *Reoviridae*. SRBSDV was found to be transmitted by white-backed planthopper (WBPH), which had not been previously recognized as a vector of any viruses. Zhou et al. (2013) summarized biological and molecular characteristics of SRBSDV, and discussed key factors of its epidemiology and current options for its control. This article should serve as a basic guide to management of this newly emerged virus, which may be widely distributed in Asia.

The yellow dwarf viruses (YDVs) belonging to the *Luteoviridae* family are the most widespread group of cereal viruses worldwide. Barley yellow dwarf viruses (BYDVs) are a group of viruses included in YDVs. BYDVs were previously placed into several strains based on the differences in their biological characteristics. Genome sequence analyses revealed that the BYDV strains are classified into either the *Luteovirus* or the *Polerovirus* genera of the *Luteoviridae* family. Krueger et al. (2013) analyzed the genome sequence of one of the BYDV strains, BYDV-RMV, showed that BYDV-RMV is a polerovirus distantly related to other YDVs, and proposed to rename it Maize yellow dwarf virus-RMV. This article affirms the etiological complexity of yellow dwarf diseases of cereal crops.

A majority of cereal viruses are disseminated among plants by insect vectors. Therefore, elucidation of the mechanisms of virus-insect association is essential to understand how an insect-transmitted virus completes its life cycle. Phytoreoviruses such as rice gall dwarf virus (RGDV) and rice dwarf virus (RDV) are persistent problems in East Asian countries. RGDV and RDV propagate in their vector insects before they are transmitted to plants. Miyazaki et al. (2013) provided detailed insights into the major events underlying life cycles of RGDV and RDV in the cells of their insect vectors revealed by high-resolution images obtained with advanced electron microscopy (EM) techniques such as cryo-EM and tomography. The article shows how individual viral proteins of RGDV and RDV play specific roles during entry, replication, assembly and intra- and intercellular transport of viruses in the insect vector cells.

Chen et al. (2013) described the specific role of an RGDV-encoded protein Pns11 in cell-to-cell spread of RGDV among the cells of its leafhopper vector. Cytopathologic studies conducted in the cultured leafhopper cells and non-host insect cells revealed that Pns11 is the minimal viral component of tubules mediating transport of RGDV among the insect cells. The involvement of Pns11 in tubule formation was confirmed by a double-stranded (ds) RNA-induced RNAi system, demonstrating the versatility of the RNAi system in function analyses of genes from viruses that can multiply in insect cells.

Once in a plant, a virus moves from one cell to another through the host plasmodesmata. A plant virus genome generally encodes one or more movement proteins that modify the structure of plasmodesmata for the virus to spread to adjacent cells. Because of the lack of reverse genetic systems for most rice viruses, identification of candidate genes for a movement protein of rice viruses had relied mostly on the similarities of rice virus genes to known movement protein genes. However, with development of gene *trans*-complementation systems, the genes encoding a movement protein have been experimentally identified for several rice viruses. Hiraguri et al. (2014) summarized the genome structures of individual rice viruses, and discussed the characteristics and probable functions of the genes that were experimentally confirmed or predicted to be involved in cell-to-cell movement.

In an epidemiological view, revealing the migration patterns of virus-transmitting insects is critical to decipher how virus diseases are actually spread to extensive crop production areas. Three species of planthoppers, small brown planthopper (SBPH, *Laodelphax striatellus*), BPH (*Nilaparvata lugens*), and WBPH (*Sogatella furcifera*) are widely distributed in rice-growing areas of Asia. They are economically important pests since rice plants incur direct damages from feeding of the planthoppers as well as indirect damages from viruses they transmit. SBPH transmits rice stripe virus (RSV) and rice black-streaked dwarf virus (RBSDV), BPH transmits rice grassy stunt virus (RGSV) and rice ragged stunt virus, and WBPH transmits SRBSDV. Otuka (2013) provided an overview of migration patterns of these planthoppers in Asia simulated based on the results from the long-term spatio-temporal trajectory analyses of their movement. The article showed that recent outbreaks of rice virus diseases in Asia can be largely accounted for by the long-distance migration of the planthoppers, and suggests that a reliable forecasting system for rice virus diseases can be established by monitoring the migration of the vector insects.

Rapid and accurate detection of viruses in host plants and insect vectors is an essential procedure in efficient management of cereal viruses. Uehara-Ichiki et al. (2013) reviewed the current technologies available for detection of rice viruses in plants and insect vectors. Serology-based methods such as enzyme-linked immunosorbent assay and dot-immunobinding assay had been developed for rice viruses, and have been adopted mainly in places where a large number of samples needed to be processed. Since the genome sequences of most rice viruses have been determined, most rice viruses also can be identified by nucleic acid-based methods, which are generally more sensitive than serology-based methods. This article addressed the strengths, and the limitations of each detection technique and it will be helpful for researchers to consider the best option for their needs.

Exploitation of natural resistance genes against cereal viruses is one of the most practical ways to manage cereal viruses in fields. However, the sources of natural resistance genes are very limited or lacking for many viruses and the durability of some natural resistance genes is often questionable. To overcome such limitations, many attempts have been made to invent artificial resistance to cereal viruses. Sasaya et al. (2014) described development of transgenic rice plants that showed a high level of resistance to phytoreoviruses (RDV and RGDV), a fijivirus (RBSDV), and tenuiviruses (RGSV and RSV) using a viral dsRNA-induced RNAi technology. Examination of extensive sets of transgenic plants expressing a dsRNA fragment specific to one of the genes of these viruses showed that the reactions of the transgenic plants to the targeted viruses varied depending on the gene-specific dsRNA expressed. Overall, their observations suggested that target virus genes should be carefully selected to attain a high level of virus gene-derived resistance.

Accumulation of vast amounts of information on plant genomes and development of high-throughput gene expression analysis technologies have expedited research efforts aiming to reveal the diverse molecular responses of host plants to viral pathogens. This research topic includes three articles which addressed the host gene responses associated with cereal virus infection. NAC transcription factors are a large family of plant transcriptional regulators, which are involved in various development processes and tolerance responses to biotic and environmental stresses. Nuruzzaman et al. (2013) reviewed the expression patterns of NAC transcription factor genes in responses to environmental stresses and plant pathogens including cereal viruses such as RDV and RSV. They proposed the possible scenarios on how particular NAC transcription factors regulate downstream genes which were known to be involved in defense and tolerance responses to biotic and environmental stresses.

Rice tungro spherical virus (RTSV) and rice tungro bacilliform virus synergistically interact and cause a serious disease in rice, although RTSV alone rarely induces any discernible symptoms in Asian rice (*Oryza sativa*). Budot et al. (2014) showed that RTSV, which is recognized as a latent virus in Asian rice, can cause serious stunting in African rice (*O. glaberrima*), and the degree of stunting in African rice appeared to be associated with suppression of particular cell wall-related genes, which were also suppressed in Asian rice plants stunted by infection with other rice viruses.

RGSV has been a serious threat to rice cultivation in Southeast Asian countries in recent years. Typical symptoms of RGSV are stunting and excessive tillering. Satoh et al. (2013) analyzed the gene expression profile of a rice plant infected with RGSV to reveal the gene expression patterns associated with the typical symptoms. They concluded that excessive tillering of rice infected with RGSV might be related to activation of genes involved in inactivation of gibberellin and auxin, and suppression of genes for strigolactone signaling, while stunting might be associated with suppression of genes related to synthesis of cell walls and chlorophyll.

## Conflict of interest statement

The authors declare that the research was conducted in the absence of any commercial or financial relationships that could be construed as a potential conflict of interest.
